# A sample preparation workflow for adipose tissue shotgun proteomics and proteogenomics

**DOI:** 10.1242/bio.036731

**Published:** 2018-10-25

**Authors:** Jane I. Khudyakov, Jared S. Deyarmin, Ryan M. Hekman, Laura Pujade Busqueta, Rasool Maan, Melony J. Mody, Reeti Banerjee, Daniel E. Crocker, Cory D. Champagne

**Affiliations:** 1 Department of Biological Sciences, University of the Pacific, Stockton, CA, USA; 2 Conservation and Biological Research Program, National Marine Mammal Foundation, San Diego, CA, USA; 3 Department of Biology, Sonoma State University, Rohnert Park, CA, USA

**Keywords:** Adipose, Blubber, Proteomics

## Abstract

Animals with large adipose stores, such as marine mammals, may provide insights into the evolution and function of this multifunctional tissue in health and disease. In the absence of sequenced genomes, molecular information can be rapidly obtained by proteomics and transcriptomics, but their application to adipose tissue is hindered by low nucleic acid and protein yields. We sequenced and compared proteomes isolated from the blubber of four elephant seals using phenol and guanidine thiocyanate (Qiazol) or detergent (sodium deoxycholate) buffer. Qiazol recovered more subcellular proteins such as metabolic enzymes, in addition to extracting RNA, facilitating proteogenomic analyses of small lipid-rich tissue biopsies. We also compared proteomics data analysis platforms and found that *de novo* peptide sequencing improved protein identification sensitivity compared to database search alone. We report sample preparation and data analysis workflows for proteogenomics and a proteome of elephant seal blubber containing 2678 proteins, including many of interest for further functional studies.

This article has an associated First Person interview with the first author of the paper.

## Abstract (precis)

**Summary:** Proteins that are compatible with shotgun proteomics can be isolated from small amounts of adipose tissue at the same time as RNA, facilitating proteogenomics studies in non-model animals.

## INTRODUCTION

Adipose is a complex organ that participates in energy storage, thermogenesis, immunity and regulation of metabolic homeostasis. Specialized fat deposits arose early in the metazoan lineage and have supported evolutionary adaptations such as migration, hibernation and lactation, among others (Birsoy et al., 2013; Pond, 2017). While adipose tissue has been studied extensively in humans and laboratory animals due to the emergence of a global obesity epidemic, non-model organisms can provide fundamental information on metabolic adaptations in animals and potentially novel insights into mechanisms by which adipose function is dysregulated in disease (Grabek et al., 2015; Houser et al., 2013). Such insights can be rapidly obtained via non-targeted approaches such as transcriptomics and proteomics in the absence of available genomes (Nesvizhskii, 2014).

With some of the largest subcutaneous adipose stores (modified as blubber) in the animal kingdom, marine mammals hold valuable information about rapid fat accrual and loss, lipid-based metabolism and the physiological effects of lipophilic pollutants (Bossart, 2011; Houser et al., 2013). Several recent studies have used omics technologies to profile gene and protein expression in blubber (Brown et al., 2017; Kershaw et al., 2018; Khudyakov et al., 2017; Van Dolah et al., 2015). However, widespread application of these approaches to marine mammal systems is hindered by the technical challenge of obtaining sufficient quantities of nucleic acids and proteins from small biopsies of lipid-rich tissues with low nuclear and cytoplasmic content. Indeed, most proteomics studies of marine mammals to date have used tissue matrices other than blubber (Neely et al., 2015a, b; Sobolesky et al., 2016).

Nucleic acids and proteins are commonly isolated from cells and tissues using separate pipelines, with phenol and guanidine thiocyanate solutions (e.g. Trizol®) used for RNA and DNA extraction and detergents (e.g. sodium dodecyl sulfate, SDS) for protein isolation (Feist and Hummon, 2015; Zhang et al., 2013). The Trizol® reagent was originally developed for simultaneous extraction of DNA, RNA, and proteins (Chomczynski, 1993), but its effectiveness for RNA and protein extraction from adipose, and proteome completeness and compatibility with tandem mass spectrometry (MS/MS), have not yet been described. In this study, we compared two lysis buffers for shotgun proteome sequencing of adipose tissue: (1) a detergent buffer containing sodium deoxycholate (SDC method), and (2) Qiazol®, a solution similar to Trizol® that was developed for nucleic acid isolation from lipid-rich samples (QIA method). We used blubber samples collected from four juvenile northern elephant seals (*Mirounga angustirostris*), a fasting-adapted marine mammal study system frequently used in comparative metabolic physiology studies, including analyses of cellular responses to stress and fasting (Khudyakov et al., 2017; Martinez et al., 2018). Marine mammal blubber is vertically stratified by fatty acid composition and function; the outer layer plays a role in thermoregulation while the inner layer is more metabolically active (Strandberg et al., 2008). We used the outer half of blubber biopsies in this study, as this layer is typically sampled by remote biopsy dart from many marine mammals (Hunt et al., 2013).

We show that a larger number of unique proteins, including those involved in metabolism and protein translation, can be identified in samples lysed using QIA with the added benefit of RNA isolation, and that *de novo* peptide sequencing (PEAKS Studio) combined with database search increases sensitivity of protein identification compared with database search alone (SEQUEST in Proteome Discoverer). We report the first elephant seal outer blubber layer proteome containing a number of metabolic enzymes and adipokines of interest to comparative physiologists and provide an optimized protocol for proteogenomics studies of adipose tissue.

## RESULTS AND DISCUSSION

### Protein yield

The study was conducted using four biological replicates of blubber tissue. Proteins were isolated from one half of each sample using the SDC method and RNA and proteins were isolated from the other half using the QIA method. SDC yielded 1.85-fold more protein than QIA (paired *t*-test, t=4.48, *P*<0.05). Mean protein yields per mg wet mass tissue for SDC and QIA were 13.7 µg/mg (s.d.=1.8) and 7.4 µg/mg (s.d.=2.2), respectively. Lower protein yields have been reported for Trizol® compared with detergent-based buffers (Yamaguchi et al., 2013). However, QIA also recovered mean 4.6 µg (s.d.=4.2) of total RNA per sample. Variability in RNA yields between samples (range: 1.78–10.89 µg) could be due to decreased efficiency of homogenization and silica column-based RNA purification with higher tissue inputs. RNA isolated by QIA from blubber samples had high purity and integrity. The mean 260/280 and 260/230 ratios were 1.99 (s.d.=0.07) and 1.75 (s.d.=0.25), respectively. The mean RNA integrity number (RIN) was 7.67 (s.d.=0.39) and mean rRNA ratio was 0.81 (s.d.=0.12) (Fig. S1). RNA integrity was above the threshold (RIN 7) commonly recommended for RNA sequencing (Gallego Romero et al., 2014).

### Protein identification

**Fig. 1.**
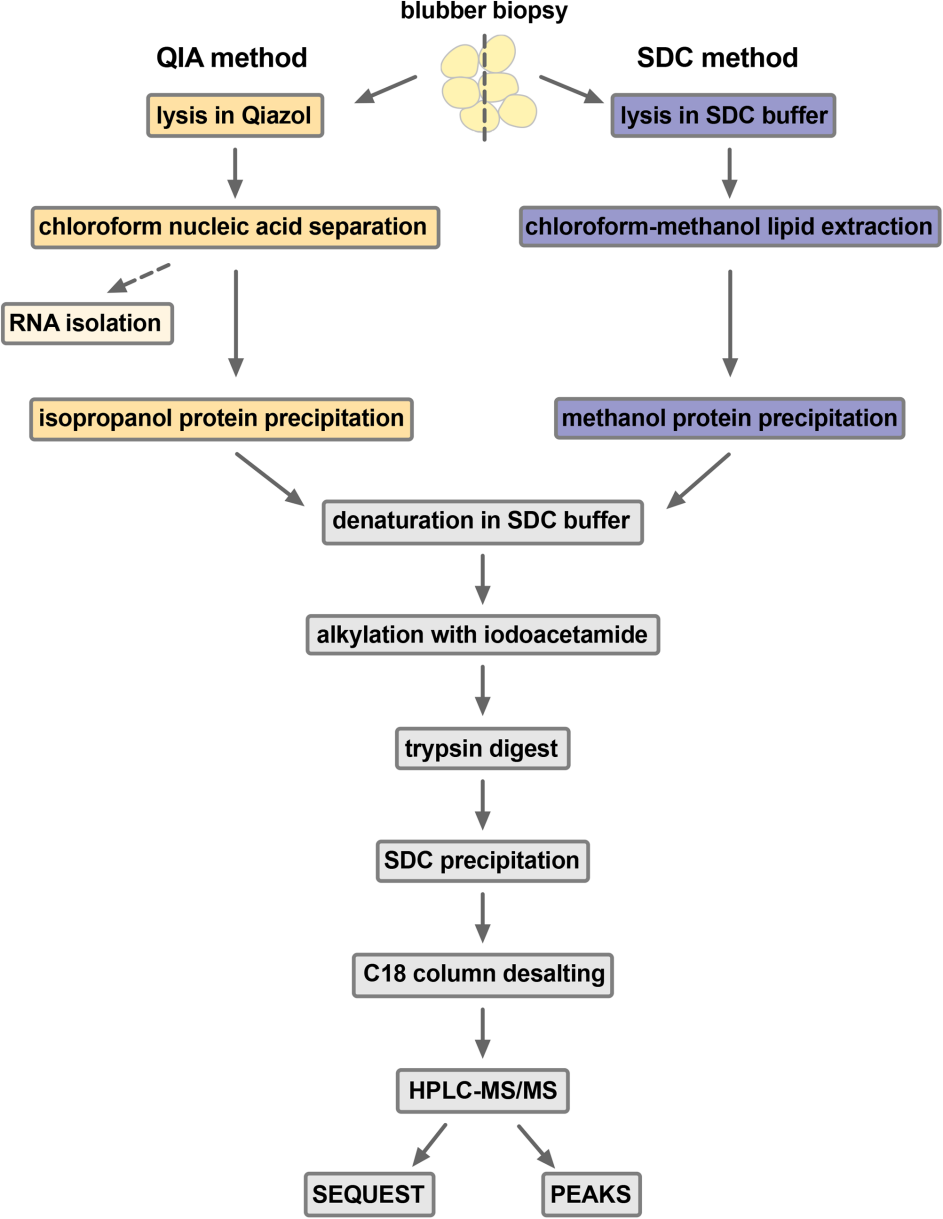
**Sample preparation workflows used in the study.** Elephant seal blubber samples were divided in half and each was lysed by bead beating with either Qiazol® (QIA method) or buffer containing sodium deoxycholate detergent (SDC method). After protein precipitation from tissue lysates, samples were treated identically (grey boxes). MS/MS data was analyzed by SEQUEST database search in Proteome Discoverer or by *de novo* sequencing and PEAKS DB database search in PEAKS Studio.

**Fig. 2.**
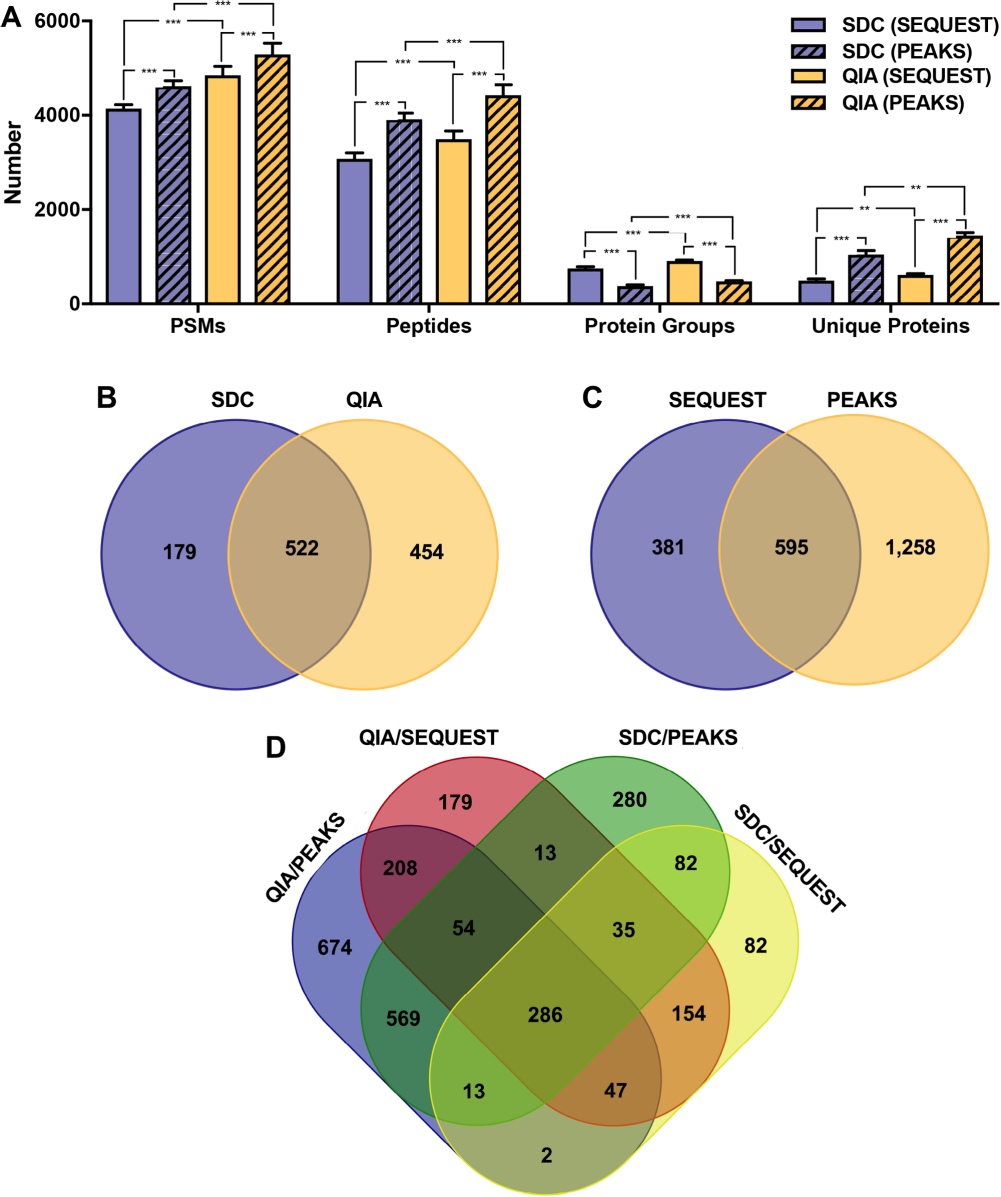
**Comparison of proteins identified using two sample preparation and two data analysis workflows.** (A) Numbers of peptide spectrum matches (PSMs) and peptides, protein groups, and unique proteins (with two or more unique peptide hits) identified by SEQUEST or PEAKS in samples prepared using SDC and QIA. SwissProt (2/13/2018) database was used for searches and only hits with false discovery rates (FDR) <1% were retained. Asterisks denote significant differences between sample preparation methods and software platforms: ***P<0.0001, **P<0.001. Sets of identified proteins were compared between (B) SDC and QIA methods and SEQUEST database search, (C) SEQUEST and PEAKS protein identification methods for QIA samples, and (D) between each sample preparation method and software platform.

After processing using standard methods [Fig. 1; Bodzon-Kulakowska et al. (2007)], protein samples were analyzed by HPLC-MS/MS, producing mean 26,621 (s.d.=1031) MS/MS spectra for SDC samples and mean 25,712 (s.d.=1374) MS/MS spectra for QIA samples. We first performed peptide spectrum matching and a SwissProt database search using SEQUEST in Proteome Discoverer. SEQUEST identified 13.3% and 16.1% of all MS/MS spectra from SDC and QIA samples, respectively, which is within the range of 10–30% reported in the literature (Houel et al., 2010). QIA samples had 1.17-fold more peptide spectrum matches (PSMs; *F*_1,10_=100.45, *P*<0.0001), 1.13-fold more peptide groups (*F*_1,10_=67.01, *P*<0.0001), 1.21-fold more protein groups (*F*_1,10_=59.09, *P*<0.0001), and 1.25-fold more unique proteins (with two or more unique peptide hits; *F*_1,10_=24.54, *P*<0.001) than SDC samples (Fig. 2A). Therefore, while total protein yields were lower with QIA, this method produced more identified peptides and proteins than SDC. We then repeated the SEQUEST search with a custom database of a translated elephant seal blubber transcriptome (Khudyakov et al., 2017). QIA-isolated samples had 6048 PSMs (s.d.=373), 4620 peptide groups (s.d.=383), 974 protein groups (s.d.=53) and 647 unique proteins (s.d.=45), while the SDC-isolated samples had 5473 PSMs (s.d.=172), 4348 peptide groups (s.d.=174), 865 protein groups (s.d.=46) and 564 unique proteins (s.d.=31). Numbers of PSMs and proteins were significantly different between methods (PSMs: *F*_1,3_=17.16, *P*<0.05; protein groups: *F*_1,3_=18.52, *P*<0.05; unique proteins: *F*_1,3_=16.33, *P*<0.05). Therefore, we identified hundreds of proteins predicted from the elephant seal transcriptome by MS/MS, validating our transcriptome and proteome methods and providing a workflow for proteogenomics. However, since the translated transcriptome was annotated by BLASTP using the same database, we focused subsequent functional analyses on proteins identified directly by SEQUEST SwissProt database search.

### Functional annotation of proteins

**Fig. 3.**
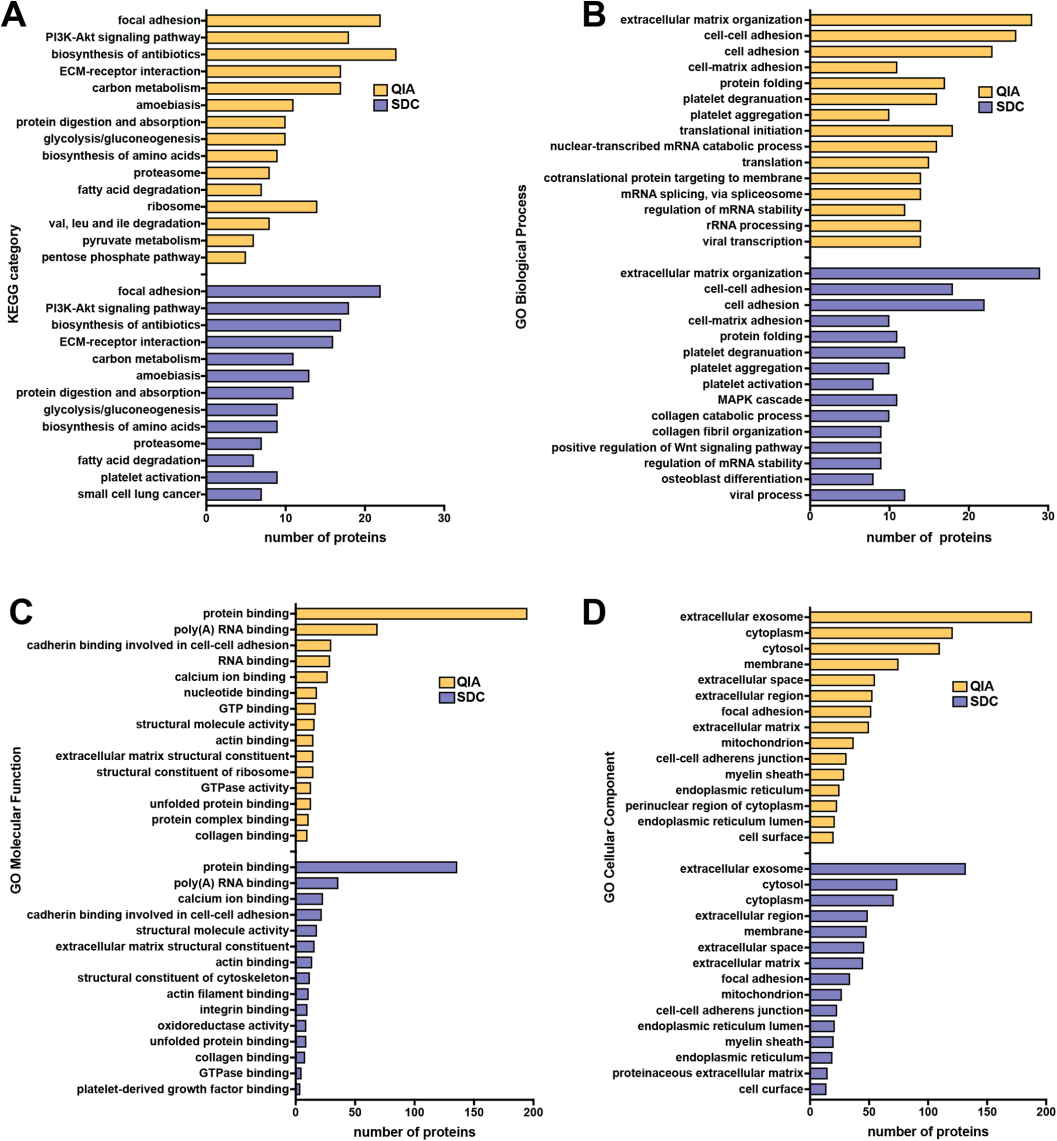
**Comparison of functional categories enriched in protein datasets obtained using two sample preparation workflows.** Top (A) KEGG pathways and (B) gene ontology (GO) biological process, (C) molecular function, and (D) cellular component categories that were significantly overrepresented (adjusted *P*<0.05) in the elephant seal blubber proteome isolated using either QIA (yellow) or SDC (blue) methods, relative to the entire human genome.

To facilitate functional analyses, we repeated SEQUEST SwissProt database search with biological replicates concatenated as ‘fractions’ into one pooled sample for each QIA and SDC. We identified 976 and 701 proteins from pooled QIA and SDC samples, respectively (2.5-fold more unique proteins with QIA than SDC; Fig. 2B). The top 5 (by number of proteins) KEGG categories overrepresented in both datasets were focal adhesion, PI3K-Akt signaling pathway, biosynthesis of antibiotics (which includes many enzymes involved in lipid metabolism), ECM-receptor interaction, and carbon metabolism (Fig. 3A). KEGG categories enriched uniquely in the QIA dataset were ribosome, pyruvate metabolism, pentose phosphate pathway, valine, leucine and isoleucine degradation, while those unique to SDC were platelet activation and small cell lung cancer. Therefore, the QIA method recovered more proteins involved in metabolism and protein synthesis than SDC.

We identified 37 and 41 GO biological process (BP) categories enriched in the QIA and SDC datasets, respectively (Fig. 3B). The top overrepresented categories in both datasets were associated with cell-matrix interactions and protein folding. BP categories unique to the QIA dataset included translation, mRNA splicing, rRNA processing, gene expression, pentose-phosphate shunt and response to calcium ion. BP categories that were enriched only in the SDC dataset included osteoblast differentiation, epithelial cell differentiation, muscle contraction, MAPK cascade, tumor necrosis factor-mediated signaling pathway, cellular response to transforming growth factor beta stimulus, regulation of cell migration, nucleosome assembly and blood coagulation. While both methods recovered proteins involved in cell–cell and cell–matrix adhesion, QIA isolated additional proteins were involved in mRNA processing and protein synthesis, while SDC recovered more proteins involved in cell signaling and differentiation.

There were 22 and 16 GO molecular function (MF) categories enriched in the QIA and SDC datasets, respectively (Fig. 3C). Top MF categories for each method were protein and RNA binding and cadherin-mediated cell–cell adhesion. MF categories enriched only in the QIA dataset were nucleotide binding, structural constituent of ribosome, protein complex binding, heparin binding and chaperone binding. MF categories unique to the SDC dataset were oxidoreductase activity and platelet-derived growth factor binding. Therefore, while representation of molecular functions was similar in both protein datasets, some proteins with oxidoreductase activity of interest to marine mammal physiology (e.g. fatty acid synthase, peroxiredoxin, alcohol dehydrogenase) were not isolated by QIA.

We identified 50 and 48 GO cellular component (CC) categories that were enriched in the QIA and SDC datasets, respectively (Fig. 3D). Top CC categories for both methods were extracellular exosome, cytoplasm/cytosol, membrane, and extracellular space/matrix. CC categories enriched only in the QIA dataset included perinuclear region of cytoplasm, mitochondrial matrix, ribosome, extrinsic component of membrane, spliceosomal complex and nuclear matrix. CC categories enriched only in the SDC dataset were actin cytoskeleton, protein complex, cell–cell junction, lysosomal lumen, sarcolemma, lipid particle, smooth endoplasmic reticulum and lamin filament. The large number of extracellular proteins recovered by both methods is consistent with thick basal lamina and abundant connective tissue proteins characteristic of adipose tissue (Mariman and Wang, 2010). The abundance of extracellular vesicle (EV)-related proteins recovered by both methods is interesting due to their potential role in regulation of metabolism and immunity in adipose tissue (Gao et al., 2017). However, the QIA method isolated more subcellular proteins than SDC, including those associated with the nucleus and mitochondria, suggesting that it may be more efficient at solubilizing intracellular membranes than detergent.

### *De novo* peptide sequencing

We performed *de novo* peptide sequencing combined with PEAKS DB SwissProt database search (Zhang et al., 2012) to evaluate whether this approach would increase sensitivity of peptide/protein identification. PEAKS produced 4534 (s.d.=567) and 4443 (s.d.=671) *de novo*-only spectra for samples prepared using SDC and QIA, respectively. For both sample preparation methods, PEAKS had approximately 1.10-fold more PSMs (*F*_1,10_=44.27, *P*<0.0001) and identified 1.27-fold more peptides (*F*_1,10_=242.24, *P*<0.0001) and 2.24-fold more unique proteins (*F*_1,10_=173.73, *P*<0.0001) than SEQUEST (Fig. 2A). However, the number of protein ‘groups’ identified by PEAKS was approximately 1.95-fold lower than SEQUEST (*F*_1,10_=572.21, *P*<0.0001) due to differences in protein isoform clustering approaches (Paulo, 2013). Therefore, combined *de novo* peptide sequencing and PEAKS DB database search had greater sensitivity than a SEQUEST database search alone, as previously reported (Zhang et al., 2012).

Lastly, we compared the sets of unique proteins identified by SEQUEST and PEAKS using the SwissProt database in the pooled QIA samples. The two software platforms identified a common set of 595 proteins; an additional 1258 were identified only by PEAKS and 381 were identified only by SEQUEST (Fig. 2C). Different protein identification approaches and database search algorithms are known to produce different sets of proteins from the same mass spectra, even with identical search parameters (Paulo, 2013; Russeth et al., 2006). Integrating results from multiple search engines can increase the number of identified proteins and validate those that are commonly identified by different algorithms, and several bioinformatics tools have been developed for this purpose (Paulo, 2013).

Overall, we identified overlapping, but distinct sets of proteins from blubber samples using two different sample preparation methods and two different MS/MS data analysis platforms (Fig. 2D). Proteins of interest to the metabolic and comparative physiology communities identified in the study include fatty acid transporters, lipid droplet proteins, and lipid metabolism enzymes (Table S3). In total, we identified 2678 proteins from the outer blubber of northern elephant seals, of which 286 were common to all four pipelines used in the study. Differences in numbers and types of proteins isolated by the QIA and SDC methods may be attributed to different membrane solubility efficiencies – reagents such as Trizol® may be more efficient than detergents at removing lipids and carbohydrates to liberate proteins (Kirkland et al., 2006). However, some have suggested that tissue lysis in phenol and guanidine thiocyanate may also lead to loss of highly hydrophobic proteins, an important consideration for adipose proteomics (Butt et al., 2007; Kirkland et al., 2006). This may be improved by optimizing solubilization conditions for proteins precipitated after Trizol® or Qiazol® extraction (Kopec et al., 2017).

## CONCLUSIONS

We found that blubber tissue lysis in Qiazol® increased the total number of identified proteins and enabled simultaneous isolation of high-quality RNA from the same tissue sample – significant advantages for researchers working with small quantities of tissue and for those interested in proteogenomics. Moreover, QIA recovered more subcellular proteins, including proteins involved in metabolism and protein synthesis, than SDC. We also found that sensitivity of protein identification in a non-model organism could be significantly improved using PEAKS *de novo* peptide sequencing in combination with a database search. Proteins identified in this study may be of interest for functional studies of marine mammals and other species in which large adipose stores play key roles in physiology.

## MATERIALS AND METHODS

### Materials

All chemicals were proteomics grade and purchased from VWR Life Science/Amresco (USA) or Thermo Fisher Scientific (USA), unless otherwise indicated.

### Sample collection

All animal handling procedures were approved by University of the Pacific and Sonoma State University Institutional Animal Care and Use Committees and conducted under National Oceanic and Atmospheric Administration Fisheries Permit No. 19108. Four juvenile (∼0.8-year old) female northern elephant seals (*M. angustirostris*) were sampled at Año Nuevo State Reserve (San Mateo County, CA, USA) in October 2017. Animals were chemically immobilized as previously described (Khudyakov et al., 2017). Blubber samples were collected from the posterior flank of the animal using a sterile 6.0 mm diameter biopsy punch (Miltex, USA), blotted on sterile gauze to remove blood, and separated into two halves: an inner (closest to muscle) blubber half and an outer (closest to skin) blubber half. Samples were placed into plastic cryogenic vials (Corning, USA), flash-frozen in liquid nitrogen, stored on dry ice, and transferred to a −80°C freezer upon return to the laboratory.

### Sample preparation

Only the outer blubber half of each biopsy was used for this study (inner blubber was used for a separate study). Each outer blubber sample was weighed and minced into small pieces, which were randomly divided into two portions of approximately 100 mg each. One portion of each sample was used for protein extraction using the detergent method (mean wet mass 97.5 mg, s.d.=8.6), while the other was used for RNA and protein extraction using the Qiazol® method (mean wet mass 102.8 mg, s.d.=9.0).

### Protein extraction using detergent (SDC method)

The SDC method of protein extraction was adapted from a previously published protocol (Pasing et al., 2017). Blubber was processed in two batches of ∼50 mg, which were minced with a sterile scalpel on ice and added to 500 µl SDC Lysis Buffer [1% w/v SDC, 8 M urea, 5 mM dithiothreitol (DTT) in 50 mM ammonium bicarbonate] in a Navy RINO^®^ bead tube (Next Advance Inc., USA). Samples were homogenized in the Bullet Blender Storm^®^ instrument (Next Advance Inc., USA) for two cycles of 2 min each at power 10, with 1 min of cooling on ice between cycles. Homogenates were further disrupted by sonication for three cycles, 15 s each, at 4 watts using a hand-held sonicator (VirSonic 60, Virtis, USA) and centrifuged to pellet insoluble cell debris and separate lipids. Tissue homogenates were extracted from under the top lipid layer and transferred to clean tubes. To remove any remaining lipids, four volumes of methanol and one volume of chloroform were added to homogenate aliquots, mixed, and centrifuged. The top layer containing lipids was removed, and four volumes of methanol were added to precipitate proteins. Protein pellets were air dried after centrifugation.

### Protein and RNA extraction using phenol-chloroform (QIA method)

The QIA method of protein extraction was adapted from the Chomczynski protocol (Chomczynski, 1993). Approximately 100 mg of blubber were minced with a sterile scalpel on ice and added to 500 µl of Qiazol® reagent (Qiagen, USA) in a Navy RINO® RNase-free bead tube (Next Advance, USA). Homogenization was conducted in the Bullet Blender Storm® (Next Advance, USA) as described above. An additional 500 µl of Qiazol® reagent was added to each tube and incubated for 5 min at room temperature with occasional vortexing. Homogenates were further disrupted using a syringe and 21-gauge needle and centrifuged to pellet insoluble cell debris and separate lipids. The homogenate was extracted from under the top lipid layer and transferred to clean tubes with 200 µl chloroform, vortexed to mix, and centrifuged at maximum speed for 15 min at 4°C to separate phases. The aqueous layer containing RNA was transferred to a clean tube and RNA purification was performed using RNeasy® Lipid Tissue Mini Kit (Qiagen, USA) (Khudyakov et al., 2017). After aqueous phase and interphase extraction, 300 µl of 100% ethanol was added to the organic phase and centrifuged to precipitate DNA. The supernatant was split into two microcentrifuge tubes and each was incubated for 10 min with 750 µl of 100% isopropanol and centrifuged again to pellet proteins. The pellets were washed twice for 20 min with 1 ml of 0.3 M guanidine hydrochloride in 95% ethanol, once with 1 ml of 100% ethanol and air dried. If not processed the same day, protein pellets were kept at −80°C in guanidine wash solution.

### Protein denaturation, digestion, and desalting

Proteins isolated by SDC and QIA methods were treated identically after precipitation. Pellets were resuspended in 250 µl of Denaturing Buffer (1% w/v SDC, 8 M urea, 5 mM DTT in 50 mM ammonium bicarbonate) by homogenization with a 21-gauge needle and continuous vortexing for 1 h at room temperature. Protein samples were then incubated at 37°C for 1 h in Denaturing Buffer, followed by alkylation with 15 mM iodoacetamide in the dark at room temperature for 30 min. Alkylation was quenched by addition of DTT to 5 mM final concentration. Samples were diluted with 50 mM ammonium bicarbonate to reduce urea concentration to <2 M and protein concentration was estimated by bicinchoninic acid assay (BCA assay) as described below. In-solution trypsin digest was conducted for 14–16 h at 37°C using Trypsin Gold^®^, Mass Spectrometry Grade (Promega) at 1:50 of µg enzyme to µg protein. Samples were acidified to pH<2 with trifluoroacetic acid to precipitate detergent and desalted using Pierce C18 Spin Columns (Thermo Fisher Scientific). To maximize protein retention, the flow-through after the addition of the sample to the column was passed back over the column twice. Proteins were eluted with 70% acetonitrile, diluted 1:1 with HPLC-grade water, lyophilized, and resuspended in 0.1% formic acid in LC/MS-grade water. Peptide concentration was estimated by BCA assay as described below.

### BCA assay

Protein and peptide concentration was estimated using Pierce BCA Protein Assay Kit (Thermo Fisher Scientific). Samples were diluted 1:10 in 50 mM ammonium bicarbonate and each sample was used in triplicate in the BCA Assay. The mean coefficient of variation (CV) for triplicates was 1.57%. Prism7 (GraphPad, USA) was used to fit the curve (third-order polynomial fit, r^2^=0.99) and extrapolate unknown sample concentrations.

### RNA quantity and quality assessment

RNA yields were determined using RNA BR Assay on the Qubit 3.0 fluorometer (Life Technologies). RNA quality was evaluated by NanoDrop spectrophotometry and microcapillary gel electrophoresis (RNA 6000 Pico assay, Bioanalyzer 2100 instrument, Agilent, USA).

### HPLC-MS/MS

Peptide samples were diluted to 150 ng/µl in 0.1% formic acid in LC/MS-grade water and 5 μl were loop injected by a Dionex Ultimate 3000 autosampler onto a reversed-phase trap column (Acclaim^®^ PepMap^®^ 100 C18 LC column; 75 µm i.d.×2 cm, 3 µm particle size, 100 A pore size, Thermo Fisher Scientific), and eluted onto a reversed-phase analytical column (EASY-Spray^®^ C18 LC column; 75 µm i.d.×15 cm, 100 A, Thermo Fisher Scientific) held at 35°C. Solvents A and B were 0.1% formic acid in water and in acetonitrile, respectively. Solvent B was used at the following concentrations: 2% for 5 min, 2–22% over 70 min, 22–38% over 25 min, 38–95% over 5 min, 95% for 5 min, return to 2% over 5 min, 2% for 25 min. Flow rates were held at 300 nl/min with each sequencing run set to 140 min. Mass spectrometry analysis was performed using Orbitrap Fusion^®^ Tribrid^®^ mass spectrometer equipped with EASY-Spray^®^ ion source (Thermo Fisher Scientific) operated in a data dependent acquisition (DDA) mode by Xcalibur 4.0 software (Thermo Fisher Scientific). Briefly, full MS1 scans were resolved by the orbitrap, and ions were selected for MS2 using DDA (charge state: 2–7; intensity threshold: 25,000). These precursor ions were quadrupole filtered and subsequently fragmented using stepped collision HCD at 28%±3 collision energy. MS2 product ions were resolved by the orbitrap. Instrument and data acquisition settings are presented in Table S1.

### MS/MS data analysis

MS/MS data was analyzed using Proteome Discoverer v2.2 (Thermo Fisher Scientific) and PEAKS Studio v8.5 (Bioinformatics Solutions Inc., USA). Peptide spectra were searched against the entire UniProt SwissProt database (downloaded on 2/13/2018) concatenated with a common contaminant database (common Repository of Adventitious Proteins, cRAP, https://www.thegpm.org/crap/index.html) using (1) SEQUEST in Proteome Discoverer or (2) PEAKS DB after *de novo* sequencing. Search parameters are shown in Table S2. False discovery rate (FDR) was estimated by searching a reversed concatenated database in Proteome Discoverer and by the ‘decoy-fusion’ approach in PEAKS (Zhang et al., 2012). Results were filtered to retain peptides and proteins with a false discovery rate (FDR) <1% and to remove those with hits to the contaminant database. Proteins were considered ‘unique’ if they had two or more unique peptides that mapped to them. DAVID Bioinformatics Resources v6.8 (Huang da et al., 2009) server was used to identify KEGG and GO categories that were overrepresented in the seal blubber proteome relative to the entire human genome (*P*<0.05, adjusted for multiple comparisons using Benjamini correction).

### Statistical analyses

All statistical analyses were conducted using JMP 13 (SAS Institute Inc., USA). Protein yields were compared by paired *t*-test (two-tailed), assuming unequal variances. The numbers of PSMs, peptides, proteins, and protein groups were compared using linear mixed models with method (SDC or QIA) and software (SEQUEST or PEAKS) as fixed effects and sample ID as a random effect.

## Supplementary Material

Supplementary information
