# Supplementary material for: A sample preparation workflow for adipose tissue shotgun proteomics and proteogenomics
Source: Biol Open. 2018 Oct 25;7(11):bio036731. doi: 10.1242/bio.036731 (PMC6262853; doi:10.1242/bio.036731)
Supplement: Supplementary information [file biolopen-7-036731-s1.pdf]

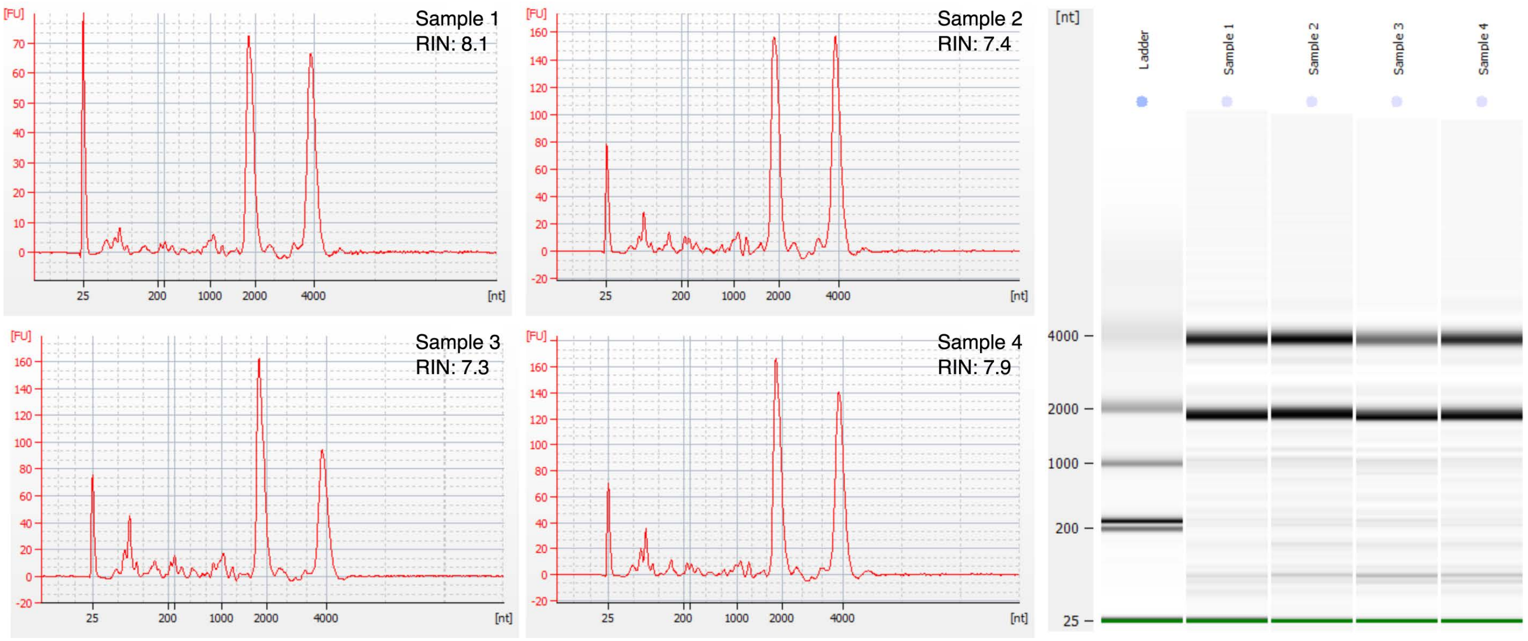

Figure S1. Microcapillary gel electrophoresis data for total RNA isolated using QIA from four elephant seal blubber samples described in the study. The electropherograms and gel image were produced using the 6000 Pico Assay on a Bioanalyzer 2100 instrument.

Biology Open • Supplementary information

Table S1. Tandem mass spectrometry (MS/MS) settings used in the study.

**MS1**	
Isolation mode	Quadrupole
Detector type	Orbitrap
Orbitrap resolution	120,000
Mass range	normal
Scan range (m/z)	300-1500 Da
RF lens	60%
AGC target	1,000,000
Max injection time	50 ms
Monoisotopic precursor	peptide
Intensity threshold	25,000
Include charge state	2-7
Dynamic exclusion after	1 time
Exclusion duration	10 sec
Mass tolerance	10 ppm
Decisions	top speed, most intense
Scan time	15-115 min
Cycle time	2 s
**MS2**	
Isolation mode	Quadrupole
Isolation window (m/z)	1.2 Da
HCD collision energy	28 ± 3%, stepped
Detector type	Orbitrap
Orbitrap resolution	15,000
Scan range mode	normal
First mass (m/z)	100 Da
AGC target	500,000
Max injection time	80 ms

Table S2. Database search parameters for peptide and protein identification.

Software	Proteome Discoverer 2.2	PEAKS v8.5
Search engine	SEQUEST	PEAKS DB
Databases	UniProt SwissProt (2/13/2018)<br>Translated blubber transcriptome (Khudyakov et al., 2017)	
Parent mass error tolerance	4.0 ppm	
Fragment mass error tolerance	0.02 Da	
Precursor mass search type	monoisotopic	
Enzyme	Trypsin, max. 2 missed cleavages	
Fixed modifications	Carbamidomethylation (C)	
Variable modifications	Oxidation (M)<br>Deamidation (NQ)<br>Max 3 mod. per peptide	
Contaminant database	CRAPome (Mellacheruvu et al., 2013)	
False discovery rate (FDR)	1%	

Biology Open • Supplementary information

Table S3. Fifteen elephant seal proteins of interest identified in the study.

UniProt Accession	Gene name	Pathway or Process
Q3Y5Z3	adiponectin (ADIPOQ)	adipocytokine signaling pathway
P16671	CD36 molecule (CD36)	adipocytokine signaling pathway
O60240	perilipin 1 (PLIN1)	regulation of lipolysis
Q96Q06	perilipin 4 (PLIN4)	PPAR signaling pathway
Q9BX66	sorbin and SH3 domain containing 1 (SORBS1)	PPAR signaling pathway
Q99685	monoglyceride lipase (MGLL)	triacylglycerol degradation
Q05469	lipase E, hormone sensitive type (LIPE)	triacylglycerol degradation
P06858	lipoprotein lipase (LPL)	lipid degradation
P02647	apolipoprotein A1 (APOA1)	cholesterol metabolism
P30084	enoyl-CoA hydratase, short chain 1 (ECHS1)	fatty acid beta-oxidation
P09110	acetyl-CoA acyltransferase 1 (ACAA1)	fatty acid metabolism
P49327	fatty acid synthase (FASN)	fatty acid biosynthesis
Q53GQ0	hydroxysteroid 17-beta dehydrogenase 12 (HSD17B12)	androgen biosynthesis
P04117	fatty acid-binding protein, adipocyte (FABP4)	lipid transport
Q8HXP2	superoxide dismutase, mitochondrial (SOD2)	superoxide metabolism

Biology Open • Supplementary information
